# Impact of 15 years of social, political and economic shocks on population mental health in the UK: a longitudinal, Bayesian quasi-experimental analysis

**DOI:** 10.1136/bmjment-2026-302688

**Published:** 2026-07-24

**Authors:** Annie Jeffery, Connor Gascoigne, Ioannis Rotous, Xuewen Yu, Sara Geneletti, Gianluca Baio, Marta Blangiardo, James B Kirkbride

**Affiliations:** 1 Division of Psychiatry, UCL, London, UK; 2 Department of Epidemiology and Biostatistics, School of Public Health, Imperial College London, London, UK; 3 Department of Statistical Science, University College London, London, UK; 4 Department of Statistics, LSE, London, UK

**Keywords:** Social Sciences, Socioeconomic Factors, Mental Health

## Abstract

**Background:**

Mental health in the UK has worsened over the last 15 years, a period marked by major systemic shocks.

**Objective:**

We investigated annual changes in population-level psychological distress and the impact of five systemic shocks: the referendum on European Union membership (Brexit), two COVID-19 lockdowns, Russia’s invasion of Ukraine and the UK Government’s 2022 ‘mini-budget’.

**Methods:**

We used longitudinal survey data from the UK Household Longitudinal Study, including 87 857 between years 2009 and 2024. We used Bayesian time series models to evaluate annual changes in psychological distress and the association with each shock, including subgroup analyses by age group, sex, ethnicity, deprivation quintile and employment status.

**Outcomes:**

We found evidence of increasing psychological distress from 2009 to 2023 (+1.085 point increase in 12-item General Health Questionnaire scores; credible interval (CrI) 0.987 to 1.184). In interrupted time series models, we observed increased psychological distress immediately after the Brexit referendum (+0.117, CrI 0.029 to 0.205) and the first COVID-19 lockdown (+0.649; CrI 0.531 to 0.767), with more insidious monthly increases in psychological distress after the Russian invasion of Ukraine (+0.010, CrI 0.004 to 0.023) and the 2022 mini-budget (+0.015, CrI 0.005 to 0.034). We found considerable sociodemographic variation by age, sex, ethnicity, deprivation and employment status.

**Conclusions:**

We found that some systemic shocks had detrimental effects on population mental health in the UK, being most pronounced for the Brexit referendum and first COVID-19 lockdown.

**Clinical implications:**

Our results demonstrate that population mental health can change in response to systemic shocks. This should inform the design of responsive clinical and public mental health provision.

**WHAT IS ALREADY KNOWN ON THIS TOPIC**

- Over the past 15 years, rates of common mental disorders in the UK have increased substantially, coinciding with major societal shocks including the European Union (EU) referendum, the COVID-19 pandemic, Russia’s invasion of Ukraine and the UK Government’s 2022 mini-budget. Although increases in mental ill health have been reported following the EU referendum and the first COVID-19 lockdown, there are no quasi-experimental studies examining the impacts of multiple systemic shocks and their contribution to overall changes in mental ill health over time.

**WHAT THIS STUDY ADDS**

- Our study is the first to use quasi-experimental methods to demonstrate the cumulative contribution of the EU referendum and the first COVID-19 lockdown to the decline in mental health in the UK population from 2009 to 2024. We also demonstrate small monthly increases in psychological distress in the months following the Russian invasion of Ukraine and the UK Government’s 2022 mini-budget.

**HOW THIS STUDY MIGHT AFFECT RESEARCH, PRACTICE OR POLICY**

- Our study uses a quasi-experimental methodology to strengthen previous associational evidence to demonstrate that systemic economic and political shocks can lead to changes in population mental health. Policymakers should use such information to anticipate the likely mental health impact of potential social, economic or mental health policies on population mental health.

## Introduction

 Mental health in many high-income countries is deteriorating. In the UK, for example, there is evidence that the annual prevalence of depression in primary care has risen from 5.8% in 2012 to 13.2% in 2023,^1^ while suicide rates in England and Wales reached their highest point in almost 25 years in 2023 (11.4 deaths per 100 000 people).^2^ There is also evidence that the increase in mental ill health has been worse for younger generations and people from minoritised ethnic backgrounds.^3^

Over the past 15 years, a series of social, economic and political shocks have occurred across the world. Events such as the 2008 financial crisis and ensuing austerity measures in many countries, including the UK, marked the beginning of a prolonged period of uncertainty, economic strain and social disruption. In the UK, this included the 2016 referendum on European Union membership (‘Brexit’)—which resulted in social polarisation^4^ and marked increases in hate crimes,^5^ the COVID-19 pandemic and associated lockdowns in 2020 and 2021, Russia’s invasion of Ukraine in February 2022—which led to extreme increases in household energy prices^6^ and the financial and political turmoil triggered by the UK Government’s ‘mini-budget’ in September 2022. The culmination of these events has led to increases in the cost of living in the UK to such an extent that it is commonly referred to as the ‘Cost of Living Crisis*’*.

Previous studies have observed increases in mental ill health during periods of rapid societal change and economic hardship.^7–9^ For example, studies have consistently found increases in mental ill health and suicide in Europe during the 2008 financial crisis.^7^ In the UK and Europe, a meta-analysis of cross-sectional studies consistently found increased prevalences of depression and anxiety during the first COVID-19 lockdown in 2020, compared with the prepandemic period.^8^ One large-scale population-based study showed increases in mental ill health in the 2 years following (ie, 2017 and 2018) the 2016 Brexit referendum.^9^ None of these studies, however, have considered the relative impact of multiple systemic shocks and their contribution to the overall decline in mental ill health over time. Furthermore, evidence concerning the effect of more recent major social, economic or political events on mental ill health since the COVID-19 pandemic is lacking.

In the present paper, we evaluated the effect that social, economic and political shocks may have on population-level psychological distress. We used Bayesian quasi-experimental methods to investigate annual changes in psychological distress in the population between 2009 and 2023, and whether these changes were associated with major systemic shocks to which most people in the UK were exposed. These events included the 2016 Brexit referendum, the first and second COVID-19 lockdowns, the Russian invasion of Ukraine, and the 2022 Government’s mini-budget. From reviewing media coverage and public sector publications, we selected these shocks based on their dominance in public debates, national media attention and our a priori expectation and expertise that these were events of sufficient magnitude to plausibly affect population mental health. We also investigated whether these annual changes in population psychological distress and associations with the selected systemic shocks varied by age, sex, ethnicity, deprivation and employment status. Based on prior evidence,^3^ we hypothesised that each systemic shock would increase levels of psychological distress over time, and that these effects would be worse in younger age groups, women, people who were from minoritised ethnic backgrounds, more deprived regions of the UK and who were unemployed.^3^

## Methods

### Study design and participants

We used longitudinal data from a nationally representative panel study—the UK Household Longitudinal Study (UKHLS).^10^ Details of the UKHLS sampling design are described elsewhere.^10^ We included all participants aged 16 years and older who had complete psychological distress data for our outcome of interest from at least one of 14 waves between January 2009 and May 2024. We excluded participants who had missing data for sex and year of birth.

Special licence access to use the UKHLS data was approved by the data owner (project 233941).

### Measures

We measured psychological distress using the 12-item General Health Questionnaire (GHQ-12) between January 2009 and May 2023. The GHQ-12 (online supplemental file A1) is a well validated, widely used scale to screen for non-psychotic mental health problems indicative of psychological distress.^11^ We treated GHQ-12 scores as a continuous outcome, with a minimum value of 0 (least psychological distress) and maximum value of 36 (most psychological distress).

We modelled changes in the mean GHQ-12 score over time, based on the exact interview date when GHQ-12 was measured. First, annual trends in the mean GHQ-12 score from 2009 to 2023. Then, mean GHQ-12 scores before and after five systemic shocks that we included as exposure variables: the Brexit referendum results (24 June 2016), the first COVID-19 lockdown (26 March 2020), the second COVID-19 lockdown (6 January 2021), the Russian invasion of Ukraine (24 February 2022) and the 2022 Government mini-budget (23 September 2022).

We included the following potential confounders, given they might affect both an individual’s experience of psychological distress and their experience of social, economic and political shocks: sex, 10-year age group, ethnicity, urban dwelling, relationship status, number of children, education, presence of any health impairment, housing, deprivation (Index of Multiple Deprivation (IMD) quintile), employment and UK born (see online supplemental file A2 for full details).

We included in our models the following additional variables as random effects (full details in online supplemental file A3): local authority district to account for any residual spatial variation, time (months) to account for residual temporal trends (not included in annual change models, only in models examining systemic shocks), unique person identifier to account for within-individual correlation from repeat measures and features to account for the survey design: the primary sampling unit (based on postal code within which sampling was performed) and sampling strata (used for stratified sampling design). This approach to account for survey design (ie, the inclusion of parameters for stratification and clustering in the modelling framework) follows the approach used in other studies evaluating the impact of government policy using UKHLS data.^12^

We imputed missing covariate data using multiple imputation by chained equations (see online supplemental file A3 for further details). We reported the proportion of missingness for each variable (table 1).

**Table 1:** Participant baseline characteristics

Total sample size	87 857
Age in years, median (IQR)	40 (26–57)
Age years group, n (%)	
Age 16–24 Age 25–34 Age 35–44 Age 45–54 Age 55–64 Age 65–74 Age 75+ Missing	20 273 (23.07) 14 663 (16.90) 15 011 (17.09) 13 267 (15.10) 11 081 (12.61) 8159 (9.29) 5403 (6.15) 0 (0.00)
Sex, n (%)	
Female Male Missing	47 403 (53.95) 40 454 (46.05) 0 (0.00)
Ethnicity, n (%)	
Asian Other Asian South Asian Black African Black Caribbean Mixed Other White British White Other Missing	1741 (1.98) 8549 (9.73) 2563 (2.92) 1740 (1.98) 1891 (2.15) 975 (1.11) 65 393 (74.43) 4885 (5.56) 120 (0.14)
UK born, n (%)	
Yes No Missing	64 784 (70.32) 14 702 (16.73) 11 371 (12.94)
Marital status, n (%)	
In relationship Not in relationship Missing	37 737 (42.95) 50 045 (56.96) 75 (0.09)
Number of own children in household, median (IQR) Missing, n (%)	0 (0–1) 0 (0.00)
Number of people in household, median (IQR) Missing, n (%)	3 (2–4) 0 (0.00)
Highest qualification, n (%)	
Higher degree Degree A-level equivalent GCSE equivalent Other None Missing	8720 (9.93) 18 585 (21.15) 17 994 (20.48) 20 741 (23.61) 7492 (8.53) 11 938 (13.89) 2387 (2.72)
Job status, n (%)	
Paid employment Self employed Unemployed Retired Maternity/parental/adoption leave Family care or home Student/training/apprenticeship Longterm sick/disabled Other Missing	38 935 (44.32) 5929 (6.75) 5842 (6.65) 15 148 (17.24) 650 (0.74) 4999 (5.69) 12 699 (14.45) 2877 (3.27) 757 (0.86) 21 (0.00)
Housing status, n (%)	
Owned outright Owned with mortgage Local authority/housing association rented Private rented Others Missing	23 655 (26.92) 33 812 (38.49) 15 604 (17.76) 12 916 (14.70) 1277 (1.45) 593 (0.67)
Health condition(s), n (%)	
Yes No Missing	27 867 (31.72) 59 937 (68.22) 53 (0.06)
Urban dwelling, n (%)	
Yes No	68 563 (78.04) 19 294 (21.96)
Equivalised household income sextile, n (%)	
1 (highest income) 2 3 4 5 6 (lowest income) Missing	5757 (6.55) 10 867 (12.37) 14 205 (16.17) 17 503 (19.92) 23 188 (26.39) 15 266 (17.38) 1071 (1.22)
Index of Multiple Deprivation quintile, n (%)	
1 (most deprived) 2 3 4 5 (least deprived) Missing	20 406 (23.23) 18 165 (20.68) 17 312 (19.70) 16 383 (18.65) 15 591 (17.75) 0 (0.00)
Number of waves responded to, median (IQR)	4 (1–10)

A-level, Secondary education to age 18; GCSE, Secondary education to age 16.

### Statistical analysis

Modelling proceeded in several stages, as summarised below (see online supplemental file A3 for full model specifications, including Bayesian priors). First, we used a Bayesian linear regression model to estimate mean differences (MDs) and 95% credible intervals (CrIs) in GHQ-12 for each calendar year relative to our reference year, 2009.

Second, we used Bayesian interrupted time series (ITS) models to estimate MDs and 95% CrIs in GHQ-12 score before and after each systemic shock. ITS is a quasi-experimental design that can be used to evaluate causal effects of an event or the introduction of population-level policies (online supplemental file A3). The ITS design uses a time series of the outcome to establish an underlying trend, which is ‘interrupted’ by an intervention, here each systemic shock, at a known point in time (here measured in months). We used each participant’s interview date on which the GHQ-12 was assessed to assign their level of psychological distress before or after each shock. The ITS model estimated two parameters, the level change in psychological distress in the sample immediately after each shock compared with before each shock and the slope change after each shock (ie, any linear change in the rate of psychological distress over time in the months after each shock). We initially used separate ITS models for each shock due to collinearity between the multiple time variables that would have occurred if all five shocks were combined in a single ITS model. As a secondary analysis, we combined the systemic shocks in a simpler preshock/postshock comparative model (similar to an ITS but without estimation of the slope change parameter since each shock), which allowed us to determine the independence of each systemic shock on psychological distress.

To investigate possible differences by sociodemographic characteristics, we performed subgroup analyses, repeating each model (the linear regression, each ITS and the preshock/postshock secondary model), stratified by each of the following variables (defined above): sex, age group, ethnicity, IMD quintile, employment status. We removed each sociodemographic characteristic as a confounder when stratifying by it but otherwise assumed the same confounding structure across all models.

For all models, we specified minimally informative priors on all parameters (see online supplemental file A3). We fitted the model using integrated nested Laplace approximations (INLAs) through the R-INLA package, a computationally efficient approach that provides accurate approximations of the posterior distribution for all the model parameters.^13^

We performed all analyses in R V.4.3.1.

### Role of funding source

The funder of the study had no role in study design, data collection, data analysis, data interpretation or writing of the report.

## Findings

We included 87 857 participants of the UKHLS who completed at least one instance of the GHQ-12 psychological distress scale between January 2009 and May 2024 (table 1). The sample was 53.95% female, 74.43% of white British ethnic background, with a median age of 40 (IQR: 26–57); this is broadly representative of the UK population.^14^

### Changes over time

We found evidence of increasing psychological distress over time (table 2). Using 2009 as a reference year, psychological distress was relatively stable until 2015 (with the exception of a 0.230 point increase of GHQ-12 score, CrI 0.155 to 0.305 in 2013), followed by more consistent increases in psychological distress from 2016 to 2019, up to a 0.617 point increase of GHQ-12 score in 2019 compared with 2009 (CrI 0.538 to 0.696). In 2020, we observed a sharp increase in psychological distress (+1.168, CrI 1.088 to 1.247), which persisted throughout the remainder of the study period (in 2023: +1.085, CrI 0.987 to 1.184).

**Table 2:** Results showing changes in GHQ-12 score relative to time (years), systemic shocks and inflation

Change in psychological distress over time—from 2009 to 2023		
Year	Mean difference in GHQ-12 (credible interval)	
2009	Reference	
2010	0.104 (0.032 to 0.177)	
2011	0.118 (0.044 to 0.191)	
2012	0.095 (0.020 to 0.169)	
2013	0.230 (0.155 to 0.305)	
2014	0.107 (0.031 to 0.183)	
2015	0.046 (−0.030 to 0.122)	
2016	0.210 (0.134 to 0.286)	
2017	0.413 (0.336 to 0.490)	
2018	0.529 (0.451 to 0.607)	
2019	0.617 (0.538 to 0.696)	
2020	1.168 (1.088 to 1.247)	
2021	1.144 (1.062 to 1.225)	
2022	1.028 (0.946 to 1.109)	
2023	1.085 (0.987 to 1.184)	
ITS model: effect of systemic shocks on psychological distress*		
Systemic shock	Mean difference in GHQ-12 (credible interval) after systemic shock	
	Immediate effect	Change in effect over time
1: Brexit referendum	0.117 (0.029 to 0.205)	0.005 (−0.002 to 0.013)
2: COVID-19 lockdown 1	0.649 (0.531 to 0.767)	−0.029 (−0.038 to −0.020)
3: COVID-19 lockdown 2	−0.111 (−0.412 to 0.205)	−0.034 (−0.044 to −0.024)
4: Russian invasion of Ukraine	0.034 (−0.121 to 0.189)	0.010 (0.004 to 0.023)
5: Mini-budget	−0.081 (−0.216 to 0.054)	0.015 (0.005 to 0.034)
Pre/post shock model: effect of systemic shocks on psychological distress†		
	Mean difference in GHQ-12 (credible interval) after systemic shock	
Systemic shock		
1: Brexit referendum	0.141 (0.054 to 0.229)	
2: COVID-19 lockdown 1	0.467 (0.361 to 0.573)	
3: COVID-19 lockdown 2	−0.099 (−0.428 to 0.237)	
4: Russian invasion of Ukraine	0.139 (0.009 to 0.270)	
5: Mini-budget	−0.052 (−0.172 to 0.068)	

* Each shock was fitted in a different ITS model, adjusted for age group, sex, ethnic group, urban dwelling, relationship status, number of children, education level, health impairments, housing status, IMD quintile, employment status and migrant status.

† All shocks were fitted in a single preshock/postshock model, mutually adjusted for other shocks as well as the confounders listed above.

GHQ-12, 12-item General Health Questionnaire; IMD, Index of Multiple Deprivation; ITS, interrupted time series.

### Main effects of shocks on psychological distress

In our Bayesian ITS (table 2), we found evidence of a small increase in psychological distress immediately after the Brexit referendum in 2016 (GHQ-12: +0.117, CrI 0.029 to 0.205) relative to the period beforehand. We observed the largest increase in psychological distress following the first COVID-19 lockdown (from March 2020; GHQ-12: +0.649; CrI 0.531 to 0.767), though this effect gradually diminished over time (−0.03 monthly, CrI −0.04 to −0.02). We found no evidence of an immediate change in psychological distress after the second COVID-19 lockdown (January 2021), Russian invasion of Ukraine (February 2022) or the 2022 Government mini-budget (September 2022); however, we did observe evidence of monthly increases in psychological distress in the months after these events (Ukraine invasion: +0.010, CrI 0.004 to 0.023; 2022 Government mini-budget: +0.015, CrI 0.005 to 0.034).

### Stratification by sociodemographic characteristics

**Figure 1:**
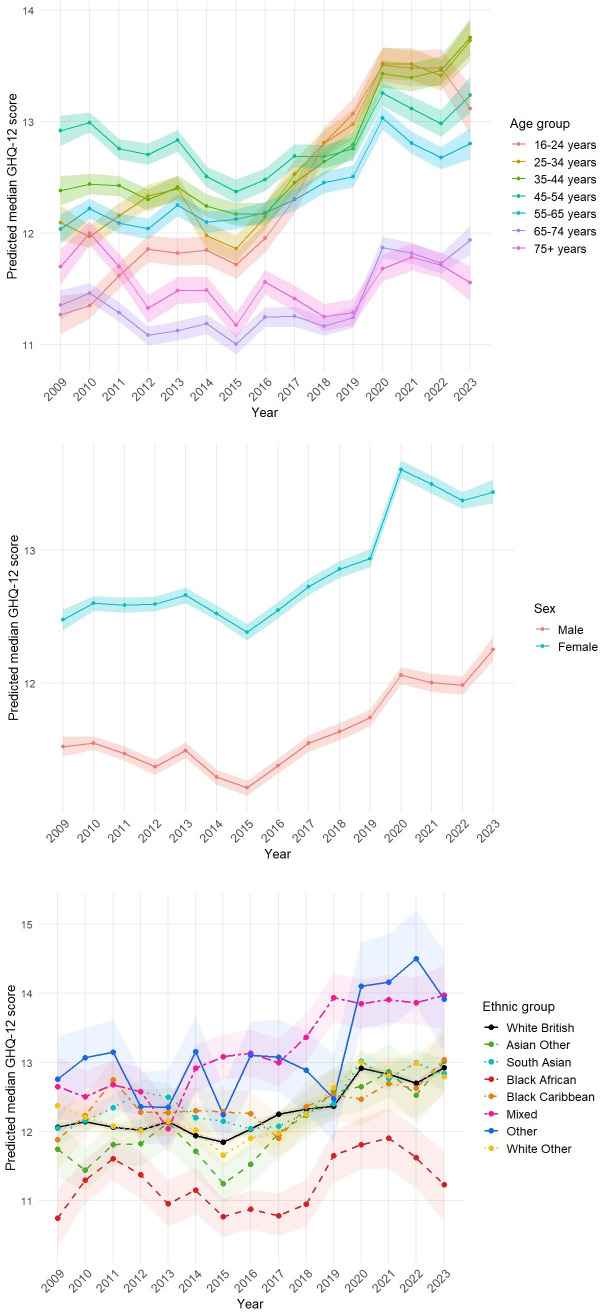
Predicted annual change in psychological distress over time, by age, sex, ethnicity. GHQ-12, 12-item General Health Questionnaire.

Young people (16–24 years) showed the greatest increase in psychological distress over the study period, peaking in 2020 (figure 1A, online supplemental file B1). Predicted psychological distress for this group was the second lowest of any age group in 2009, but highest by 2022. From 2009–2016, people aged 45–54 consistently had the worst predicted psychological distress, but from 2017 onwards we see evidence of rapid rises in psychological distress among people aged 16–44 years old who reported the worst psychological distress from 2020 onwards. People aged 65+ had less psychological distress than other age groups at all time periods, although with some evidence of worse psychological distress since 2020. Our ITS models confirmed that all age groups experienced worse psychological distress after the first COVID-19 lockdown; this change was generally stronger with older age (online supplemental file 1). We only found statistically significant evidence of an immediate effect of the Brexit referendum among those aged 45–54 (GHQ-12: +0.281, CrI 0.085 to 0.477). However, we found a gradual monthly increase in psychological distress after the Brexit referendum in people aged 16–24 (GHQ: 0.025, CrI 0.003 to 0.048) and aged 55–64 (GHQ: 0.023, CrI 0.005 to 0.041).

Women consistently experienced substantially worse psychological distress than men, which rose in both groups over the study period (figure 1B, online supplemental file B2). We found evidence that the first COVID-19 lockdown had a stronger negative effect on psychological distress for women (GHQ-12: +0.862, CrI 0.712 to 1.011) than men (+0.385, CrI 0.231 to 0.540) (online supplemental file C2).

Psychological distress tended to rise across all ethnic groups over the follow-up period (figure 1C, online supplemental file B3). People from Mixed and ‘Other’ ethnic backgrounds consistently experienced the worst predicted psychological distress, while people from black African backgrounds consistently experienced least psychological distress. Our ITS found that the Brexit referendum only had a discernible effect on worse psychological distress for people of ‘Other’ ethnicities (GHQ-12: +0.815, CrI 0.054 to 1.786; online supplemental file C3). Psychological distress worsened after the first COVID-19 lockdown in several ethnic groups, including those of white British (0.701, CrI 0.573 to 0.828), ‘White Other’ (0.571, CrI 0.212 to 0.930), South Asian (0.453, CrI 0.154 to 0.749) and ‘Other’ (1.533, CrI 0.511 to 2.539) ethnicities. For all these groups (with the exception of ‘Other’) psychological distress gradually decreased in the months after the first lockdown. We also observed evidence that psychological distress worsened considerably for the ‘Other’ ethnic group immediately after the 2022 Government mini-budget (+2.020, CrI 0.405 to 3.627).

**Figure 2:**
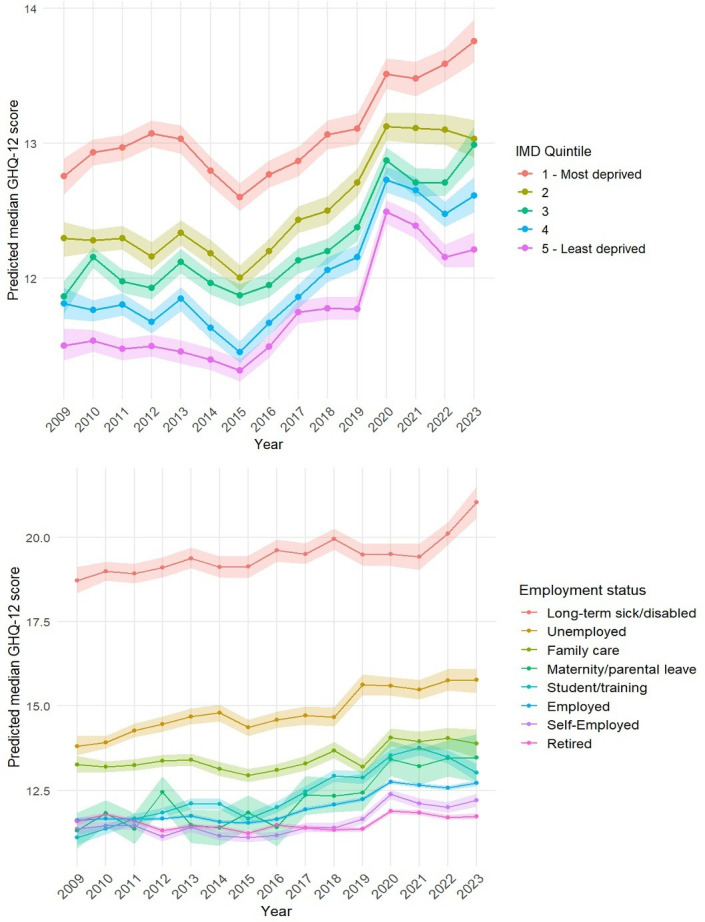
Predicted annual change in psychological distress over time, by deprivation, employment status. GHQ-12, 12-item General Health Questionnaire; IMD, Index of Multiple Deprivation.

We observed strong, dose–response relationships between greater deprivation and psychological distress that persisted over time (figure 2A, online supplemental file B4). Psychological distress inequalities by deprivation quintile were at their widest in 2013 and 2023, and narrowest in 2020, the latter effect driven by stronger increases in psychological distress in less deprived quintiles during the first COVID-19 lockdown (figure 2B, online supplemental file C4).

People who were long-term sick or disabled consistently experience the worst psychological distress, which increased over time (figure 2, online supplemental file B5), and which was followed by those who were unemployed or providing family care. Following our ITS, those who were long-term sick or disabled had the greatest increase in psychological distress immediately after the 2016 Brexit referendum (GHQ-12: +0.793, CrI 0.245 to 1.358), as well as those who were retired (GHQ-12: +0.177, CrI 0.031 to 0.322) or providing family care (GHQ-12: +0.391, CrI 0.021 to 0.771) (online supplemental file C5). We found an effect of the first COVID-19 lockdown on increasing psychological distress in most groups by employment status, except those who were unemployed or long-term sick or disabled.

### Secondary analyses

In our secondary analyses, all effects identified in our ITS models (main effects, and stratified models) remained in our preshock/postshock comparative model, which mutually adjusted for all shocks simultaneously (online supplemental file D1-5), with the single exception of the absence of an effect of the first COVID-19 lockdown previously observed in young people aged 16–24. Additionally, our secondary analyses found additional effects on worse psychological distress for the following comparisons: in the period after the Brexit referendum in all age groups (except 16–24, 35–44 and 65–74) (Supplementary File D1) and in people who were employed (Supplementary File D5); in the period after the Russian invasion of Ukraine overall (table 2; GHQ-12: +0.139, CrI 0.009 to 0.270) and in people living in the most deprived areas (Supplementary File D4) and those who were long-term sick or disabled (Supplementary File D5); and in the period after the 2022 mini budget people aged 25–34 (Supplementary File D1). We found decreases in psychological distress in the period after the 2022 mini budget in people aged 75+ (Supplementary File D1) and people of ‘White Other’ ethnicity.

## Discussion

We found that psychological distress in the UK has increased since 2009, accelerating from 2015, and with evidence that the Brexit referendum in 2016 and, to a greater extent, the first COVID-19 lockdown in 2020 contributed to these changes. Although the Russian invasion of Ukraine and 2022 Government mini-budget did not immediately lead to changes in psychological distress, we found evidence that these events were followed by insidious, although small increases in population psychological distress in the months following them; secondary analyses suggested that mental health was worse following the 2022 Government mini-budget for those living in the most deprived parts of the UK and/or who were long-term sick or disabled. Our results suggest that severe social, political and economic shocks can worsen population mental health.

Our study is consistent with increases in common mental disorders and symptoms observed in UK primary care and in non-clinical general population samples over the last decade, particularly for younger people.^3^ Although the GHQ-12 ranges from 0 to 36, small changes in the population mean may be meaningful because they can shift a substantial number of individuals across thresholds for probable common mental disorder, consistent with Geoffrey Rose’s seminal observations.^15^ For example, early analyses of the COVID-19 pandemic demonstrated that a 1-point increase in mean GHQ-12 coincided with an increase in clinically significant distress from 18.9% to 27.3%.^16^ We therefore interpret the observed changes as population-level shifts in psychological distress, rather than as individual-level clinically important differences.

We are aware of only one previous study of the effect of Brexit on population mental health, which also observed an overall increase in mental ill health in the years following the referendum.^9^ There is more consistent systematic review evidence of an effect of the first COVID-19 lockdown on immediate increases in the prevalence of depression and anxiety in the UK^8^ and throughout Europe.^17^ Consistent with this evidence, we also observed that the effect of both COVID-19 lockdowns in the UK on population mental health gradually diminished over time. However, our study shows that psychological distress did not return to prepandemic levels by the end of the study.

Previous research has suggested that the impacts of the Brexit referendum^9^ and the first COVID-19 lockdown^8^ were worst for young people, but we found little evidence to support this in our subgroup analysis by age. Interestingly, we found that psychological distress in young people (aged 16–34) had been increasing as early as 2010–6 years earlier than other age groups (figure 1A) and prior to any of the systemic shocks we examined in this paper. This means that other factors must primarily account for the robust rises in mental ill health observed in young people.^3^ During this time, austerity following the earlier global financial crisis in 2008 saw cuts to local youth services, rising child poverty and reduced economic opportunities for young people in the UK.^18^ Young people may also be vulnerable to mental health harms from various emerging socio-economic exposures, including harmful social media content^19^ and social exclusion.^20^ It is also possible that for this group and others—for whom rises in psychological distress are not fully explained by the Brexit referendum or COVID-19 lockdown—that the Brexit referendum was both a symptom and cause of growing social unrest and discontent with socio-economic opportunities in the country in the period since the 2008 global financial crisis. Anti-immigration policies,^21^ welfare reforms^12^ and reductions in local government spending^22^ during this period have all been linked to worsening mental ill health.

We demonstrated that the impact of the Brexit referendum on psychological distress was worse for people living in more deprived areas. This is consistent with previous longitudinal research showing a steeper decline in mental health in the years after the Brexit referendum for people on lower incomes.^9^ Contrary to our hypotheses, we found that psychological distress increased more steeply among employed individuals and people living in wealthier areas during the first COVID-19 lockdown. This may reflect heightened concerns about job security or changes in lifestyle, which could have amplified distress in these groups. Our work contrasts previous studies that have shown that those people with deprived socio-economic status experienced worse mental health during the pandemic,^23^ which we did not find despite observing persistently higher psychological distress in these groups over time. Consistent with existing research,^24^ we found evidence that the first COVID-19 lockdown had the greater immediate effects on psychological distress in women, older people, students and people from white and South Asian ethnic backgrounds. People who identified as long-term sick or disabled consistently reported highest levels of psychological distress across the study period, which were substantively elevated over other groups by employment status. This group experienced the greatest increase in psychological distress after the Brexit referendum, but no change in psychological distress after the first COVID-19 lockdown, for which we did not have strong a priori hypotheses. While we did not observe an overall impact of the second COVID-19 lockdown, psychological distress decreased for people who were self-employed and living in areas in the middle deprivation quintile (although, again not returning to prepandemic levels). This may indicate recovery from a previous spike in psychological distress leading up to the second COVID-19 lockdown in these groups.

Although we did not find evidence of an immediate impact of the Russian invasion of Ukraine or the 2022 mini budget on population psychological distress, these events were associated with insidious increases in psychological distress in the following months that became marked by a pronounced cost-of-living crisis.^6^ These cost-of-living impacts affected people in more deprived areas disproportionately, as we demonstrated by the widening inequalities in psychological distress observed between deprivation quintiles since 2020 (figure 2) and in the worsening psychological distress in the period after the Ukraine invasion for people living in the most deprived areas. In addition, our finding of worsening psychological distress in the long-term sick and disabled in the period after the Ukraine invasion is supported by government reports of the disproportionate impact of the cost-of-living on people who are disabled.^25^

We acknowledge some limitations in our study. First, although we used the GHQ-12, a commonly used, cross-culturally validated instrument to assess psychological distress, we recognise that a single instrument might not capture the full range of distress that people experience in relation to social, political and economic shocks—such as fear or loneliness. Future research investigating specific mental health symptoms and individual psychiatric disorders would be valuable. Second, we did not have information regarding whether an individual was personally impacted, as such we were only able to make inferences at the population level. Third, we may have misclassified the date at which a shock ‘interrupted’ population mental health, as we based this date—broadly—on policy implementation dates (the Brexit referendum, COVID-19 lockdowns, mini-budget, etc) rather than the dates at which these shocks may have induced behavioural or psychosocial responses in the population that led to psychological distress. The true onset of the effect of these shocks on population mental health may have been influenced by anticipatory or lagged effects, most plausibly in the case of the Brexit referendum where both anticipatory changes in population responses and lagged effects associated with the implementation of the referendum decision may have led to interruption misclassification. Our effect sizes should be interpreted relative to the defined interruption points, which may differ from the true onset of the effect of these shocks. Fourth, while our ITS design captured both immediate and ongoing (ie, monthly) changes in psychological distress associated with our included shocks, models were constrained to use a linear parameter to estimate monthly changes in psychological distress to the end of the follow-up period, which would not have captured non-linear changes over time. Lastly, our final two shocks—the invasion of Ukraine and the mini-budget—had limited data points (7 months) in Understanding Society between and after each event, meaning the number of participant responses for these periods was lower than for other shocks, potentially leading to underpowered analyses here.

While this study focuses on shocks that could explain the decline in population mental health, further research should also evaluate the impact of positive events, such as the Olympic Games (in UK in 2012) or the legalisation of same-sex marriage (in UK in 2014) on population-level mental health. There is some research showing that policies aimed at reducing discrimination, such as the UK 2014 legalisation of same-sex marriage, have a positive impact on mental health in minoritised groups.^26^ There is also some evidence showing that urban regeneration related to mega sporting events, such as the 2012 Olympic Games in London, can have a positive impact on mental health for people living in those areas.^27^ However, this area is under-researched using quasi-experimental designs and would provide complementary evidence to our study.

## Clinical implications

Overall, our study provides evidence that pronounced shocks that affect social and economic mobility can have pernicious effects on declining population mental health. Policymakers should use such information to ensure that social, economic and mental health policies can be put in place to both prevent further excess mental distress and treat existing elevated need appropriately. This task is considerable against continued constraints to the public purse and continued cuts to mental health service funding.^28^ Recent simulation work from Australia suggests that large economic and well-being gains could be realised by targeted reform of social, economic and health policies,^29^ and an integrated approach to tackling these problems will also be required to improve mental health in the UK.

## Supplementary material

10.1136/bmjment-2026-302688 online supplemental file 1

## Data Availability

Data may be obtained from a third party and are not publicly available.
