# Supplementary material for: Impact of 15 years of social, political and economic shocks on population mental health in the UK: a longitudinal, Bayesian quasi-experimental analysis
Source: BMJ Ment Health. 2026 Jul 24;29(1):e302688. doi: 10.1136/bmjment-2026-302688 (PMC13404486; doi:10.1136/bmjment-2026-302688)
Supplement: online supplemental file 1 [file bmjment-29-1-s001.docx]

**Supplementary File A1 – GHQ-12 questions and responses**

The next questions are about how you have been feeling over the last few weeks.

Have you recently been able to concentrate on whatever you're doing?

Option Label

1 Better than usual

2 Same as usual

3 Less than usual

4 Much less than usual

Have you recently lost much sleep over worry?

Option Label

1 Not at all

2 No more than usual

3 Rather more than usual

4 Much more than usual

Have you recently felt that you were playing a useful part in things?

Option Label

1 More so than usual

2 Same as usual

3 Less so than usual

4 Much less than usual

Have you recently felt capable of making decisions about things?

Option Label

1 More so than usual

2 Same as usual

3 Less so than usual

4 Much less capable

Have you recently felt constantly under strain?

Option Label

1 Not at all

2 No more than usual

3 Rather more than usual

4 Much more than usual

Have you recently felt you couldn't overcome your difficulties?

Option Label

1 Not at all

2 No more than usual

3 Rather more than usual

4 Much more than usual

Have you recently been able to enjoy your normal day-to-day activities?

Option Label

1 More so than usual

2 Same as usual

3 Less so than usual

4 Much less than usual

Have you recently been able to face up to problems?

Option Label

1 More so than usual

2 Same as usual

3 Less able than usual

4 Much less able

Have you recently been feeling unhappy or depressed?

Option Label

1 Not at all

2 No more than usual

3 Rather more than usual

4 Much more than usual

Have you recently been losing confidence in yourself?

1 Not at all

2 No more than usual

3 Rather more than usual

4 Much more than usual

Have you recently been thinking of yourself as a worthless person?

Option Label

1 Not at all

2 No more than usual

3 Rather more than usual

4 Much more than usual

Have you recently been feeling reasonably happy, all things considered?

Option Label

1 More so than usual

2 About the same as usual

3 Less so than usual

4 Much less than usual

Scoring: Option 1 = 0 points; Option 2 = 1 point; Option 3 = 2 points; Option 4 = 3 points. Minimum of

0 points indicates no psychological distress. Maximum value of 36 points indicates maximum

psychological distress

**Supplementary File A2 - Detailed description of confounders**

- Sex (dichotomised as male, female)
- Age in years at date of interview. Categorised into the following groups: 16-24, 25-34, 35-44, 45-54, 55-64, 65-74, 75+
- Ethnicity. Categorised as: Asian Other, South Asian, Black African, Black Caribbean, Mixed, Other, White Other, White British.
- Urban dwelling based on area of residence. Dichotomised as urban = area with population of 10,000 or more; rural = area with population less than 10,000.
- Relationship status, based on marital status. Dichotomised as in relationship (married, civil partnership, living as a couple), not in relationship (widowed, divorced, separated, never married, civil partnership, dissolved civil partnership, separated from civil partner, survived from civil partner).
- Number of children (continuous count) - total number of children aged 15 or under in the household.
- Highest educational qualification (categorised as higher degree, undergraduate degree (or equivalent), A Level (or equivalent), GCSE (or equivalent), other, none)
- Physical or mental health impairment or disability lasting more than 12 months. Dichotomised as yes or no.
- Housing status. Categorised as owned outright, owned with mortgage, privately rented, state rented (including housing association or local authority), other.
- Index of multiple deprivation (IMD) decile (the official measure of relative deprivation in England, based on 39 indicators in 7 domains – income, employment, health deprivation and disability, education and skills training, crime, barriers to housing and services, living environment. We used the most recent IMD rank based on the local authority area that the participant lived in – this included 2015 and 2019 for England, 2017 for Northern Ireland, 2016 and 2020 for Scotland, and 2014 and 2019 for Wales.
- Employment status. Categorised as employed, self-employed, unemployed, long-term sick/disabled, retired, parental leave, family care, student or in training, or other.
- UK born. Dichotomised as yes, no.
- Total net household income. Categorised into sextiles according to annual national average income published by the Office for National Statistics.

**Supplementary File A3 – Detailed description of modelling approaches**

**Multiple imputation:**

We performed multiple imputation using the MICE package in R, with 36 imputations and 10 iterations. We included in the imputation model all variables used in the analysis model (except for those used for random effects). We conducted all analyses in each imputed data set, then manually pooled the results using Rubin’s rules (C1).

**Modelling approach 1: Linear regression**

We specified our linear regression model to compare annual changes to the reference year of 2009 as follows:

*Y = β_0_*

*+ β_1_ (categorical calendar year)*

*+ β_k_ (confounders)*

*+ random spatial effect*

*+ random effect for participant identifier*

*+ random effect for primary sampling unit*

*+ random effect for sampling strata*

Random effect models and Bayesian priors are described below.

**Modelling approach 2: Interrupted time series (ITS)**

The ITS design uses a time series of the outcome to establish an underlying trend, which is ‘interrupted’ by a systemic shock at a known point in time. The effect of this shock can be evaluated as the change in the outcome before and after the shock. This quasi-experimental design has a causal interpretation because it emulates a randomised-controlled trial: if we assume that participants within each group immediately before and after the shock are exchangeable, then we can emulate a situation in which the shock was randomly allocated to those before and after (that is, that confounders, observed or unobserved, would be balanced before and after the shock). Further, the ITS design implicitly isolates the effect of the intervention from other potentially co-occurring events (C2).

We specified our ITS models as follows (each systemic shock was modelled separately):

*Y = β_0_*

*+ β_1_ (binary term for before/after shock)*

*+ β_2_ (time in months)*

*+ β_3_ (time since start of shock in months)*

*+ β_k_ (confounders)*

*+ random temporal effect*

*+ random spatial effect*

*+ random effect for participant identifier*

*+ random effect for primary sampling unit*

*+ random effect for sampling strata*

Random effect models and Bayesian priors are described below.

**Modelling approach 3: Pre-post analysis**

A pre-post design compares the outcome before and after an event. We combined binary variables for each systemic shock in one model, specified as follows:

*Y = β_0_*

*+ β_1_ (binary term for before/after Brexit referendum)*

*+ β_2_ (binary term for before/after first Covid-19 lockdown)*

*+ β_3_ (binary term for before/after second Covid-19 lockdown)*

*+ β_4_ (binary term for before/after Russian invasion of Ukraine)*

*+ β_5_ (binary term for before/after 2022 Government mini-budget)*

*+ β_6_ (time in months)*

*+ β_k_ (confounders)*

*+ random temporal effect*

*+ random spatial effect*

*+ random effect for participant identifier*

*+ random effect for primary sampling unit*

*+ random effect for sampling strata*

Random effect models and Bayesian priors are described below.

**Bayesian priors, random effects and hyperparameters:**

For all models, we specified minimally informative priors on all parameters (see Supplementary File A3). This was appropriate as the large sample size provided substantial information to identify the parameters. In this context, the priors primarily serve to stabilise estimation by propagating uncertainty in a principled way, rather than to strongly influence inference.

On the intercept, we specified a minimally informative normal prior *β_0_ ~ Normal(0, + ∞)*. On the regression parameters we specified minimally-informative, normal prior as *β_0_ ~ Normal(0, σ^2^ = 1000)*.

We used independent and identically distributed (iid) models for the random effects on individual participant identifier, sampling strata and primary sampling unit. This uses zero mean Gaussian distribution priors: *Normal(0, σ^2^)*.

Spatial random effects: We used a Besag-York-Mollie Two (BYM2) model which includes unstructured global correlations to account for similarities between Local Authorities regardless of whether they are neighbours or not, and structured local correlations capture the similarities in the response between neighbouring Local Authorities. (C3)

Temporal random effects: We used a Random Walk of order two (RW2) model to capture local correlations in time between the given year and the two years before and after. This specification is very flexible and approximates highly non-linear time trends. This random effect accounted for any residual temporal variation in psychological distress, which could be due to natural changes in time or other events that could impact mental health. (C4-5)

Random effect hyperparameters: All the standard deviations parameters for the random effects have a penalised complexity (PC) prior. (C6) PC priors are specified by *Pr(model parameters > U)=p,* where *U* is the upper bound for the distribution of the parameter and *p* is the probability of the model parameter being in the upper bound. We specified *U = 1* and *p = 0.01*. The spatial random effect also includes a mixing parameter, *φ*, which attributes how much of the spatial variation is due to the structured *(φ = 1)* or unstructured *(φ = 0)* component. We specified the PC prior for *φ* as *Pr(φ > 1/2) = 2/3* due to the prior belief that more of the spatial variation would be described by the unstructured random effect.

**References:**

C1. Enders, C. K. Applied Missing Data Analysis, Second Edition. (Guildford Press, 2020).

C2. Kim Y, Steiner P. Quasi-Experimental Designs for Causal Inference. Educ Psychol. 2016;51(34):395-405. doi: 10.1080/00461520.2016.1207177.

C3. Rue H, Held L. Gaussian Markov Random Fields. Chapter 3. 18 February 2005. New York. DOI: https://doi.org/10.1201/9780203492024

C4. Besag, J., York, J. & Mollié, A. Bayesian image restoration, with two applications in spatial statistics. Ann Inst Stat Math 43, 1–20 (1991). DOI: <https://doi.org/10.1007/BF00116466>

C5. Riebler A, Sørbye SH, Simpson D, Rue H. An intuitive Bayesian spatial model for disease mapping that accounts for scaling. Statistical Methods in Medical Research. 2016;25(4):1145-1165. DOI: <https://doi.org/10.1177/0962280216660421>

C6. Simpson D, Rue H, Riebler A, Martins T, Sørbye S. Penalising Model Component Complexity: A Principled, Practical Approach to Constructing Priors. Statistical Science, Statist. Sci. February 2017. 32(1), 1-28.

**Supplementary File B1 Yearly changes in psychological distress (GHQ-12 score) by year relative to 2009, by age group**

| **Reference value for GHQ-12 in 2009 (credible interval)**** | | | | | | | |
| --- | --- | --- | --- | --- | --- | --- | --- |
|  | **Age 16-24** | **Age 25-34** | **Age 35-44** | **Age 45-54** | **Age 55-64** | **Age 65-74** | **Age 75+** |
| **2009** | 11.95 (11.56 to 12.34) | 13.23 (12.87 to 13.60) | 13.49 (13.15 to 13.83) | 13.37 (13.04 to 13.69) | 12.04 (11.72 to 12.36) | 10.62 (10.27 to 10.98) | 10.48 (9.90 to 11.06) |
| **Mean difference in GHQ-12 relative to 2009 (credible interval)** | | | | | | | |
|  | **Age 16-24** | **Age 25-34** | **Age 35-44** | **Age 45-54** | **Age 55-64** | **Age 65-74** | **Age 75+** |
| **2010** | 0.020 (-0.208 to 0.248) | -0.090 (-0.288 to 0.108) | 0.049 (-0.124 to 0.223) | 0.111 (-0.069 to 0.291) | 0.282 (0.106 to 0.459) | 0.154 (-0.023 to 0.331) | 0.248 (0.018 to 0.477) |
| **2011** | 0.331 (0.099 to 0.562) | 0.106 (-0.094 to 0.306) | 0.128 (-0.048 to 0.303) | -0.019 (-0.201 to 0.163) | 0.155 (-0.024 to 0.333) | 0.069 (-0.110 to 0.248) | 0.170 (-0.065 to 0.406) |
| **2012** | 0.598 (0.362 to 0.833) | 0.237 (0.031 to 0.442) | 0.082 (-0.097 to 0.261) | -0.087 (-0.272 to 0.098) | 0.057 (-0.125 to 0.240) | -0.052 (-0.233 to 0.129) | -0.088 (-0.326 to 0.150) |
| **2013** | 0.665 (0.427 to 0.904) | 0.334 (0.124 to 0.544) | 0.226 (0.042 to 0.409) | 0.107 (-0.080 to 0.294) | 0.224 (0.039 to 0.410) | 0.010 (-0.173 to 0.193) | 0.088 (-0.152 to 0.329) |
| **2014** | 0.738 (0.497 to 0.979) | 0.000 (-0.214 to 0.214) | 0.059 (-0.127 to 0.245) | -0.176 (-0.364 to 0.012) | 0.113 (-0.074 to 0.299) | 0.047 (-0.137 to 0.231) | 0.067 (-0.171 to 0.306) |
| **2015** | 0.648 (0.407 to 0.890) | 0.031 (-0.184 to 0.246) | 0.080 (-0.108 to 0.267) | -0.227 (-0.417 to -0.037) | 0.073 (-0.115 to 0.261) | -0.131 (-0.316 to 0.054) | -0.150 (-0.390 to 0.090) |
| **2016** | 0.855 (0.611 to 1.098) | 0.311 (0.093 to 0.528) | 0.089 (-0.100 to 0.278) | -0.100 (-0.290 to 0.091) | 0.092 (-0.097 to 0.280) | 0.129 (-0.057 to 0.316) | 0.240 (0.000 to 0.480) |
| **2017** | 1.192 (0.941 to 1.442) | 0.634 (0.410 to 0.858) | 0.359 (0.165 to 0.554) | 0.113 (-0.082 to 0.307) | 0.241 (0.049 to 0.432) | 0.154 (-0.034 to 0.343) | 0.218 (-0.025 to 0.461) |
| **2018** | 1.603 (1.347 to 1.859) | 0.915 (0.687 to 1.143) | 0.554 (0.355 to 0.753) | 0.164 (-0.034 to 0.361) | 0.285 (0.091 to 0.478) | 0.087 (-0.103 to 0.278) | 0.138 (-0.106 to 0.383) |
| **2019** | 1.813 (1.552 to 2.073) | 1.087 (0.855 to 1.320) | 0.679 (0.476 to 0.882) | 0.227 (0.026 to 0.427) | 0.337 (0.141 to 0.533) | 0.138 (-0.055 to 0.330) | 0.154 (-0.091 to 0.398) |
| **2020** | 2.157 (1.892 to 2.421) | 1.627 (1.391 to 1.863) | 1.348 (1.140 to 1.556) | 0.749 (0.545 to 0.952) | 0.864 (0.667 to 1.061) | 0.791 (0.597 to 0.985) | 0.650 (0.404 to 0.897) |
| **2021** | 2.247 (1.972 to 2.521) | 1.639 (1.398 to 1.880) | 1.270 (1.057 to 1.484) | 0.628 (0.420 to 0.837) | 0.727 (0.526 to 0.927) | 0.795 (0.598 to 0.992) | 0.810 (0.560 to 1.059) |
| **2022** | 2.244 (1.966 to 2.523) | 1.465 (1.224 to 1.705) | 1.256 (1.042 to 1.471) | 0.444 (0.233 to 0.656) | 0.550 (0.349 to 0.752) | 0.643 (0.445 to 0.841) | 0.767 (0.518 to 1.017) |
| **2023** | 1.970 (1.645 to 2.295) | 1.678 (1.397 to 1.959) | 1.426 (1.172 to 1.680) | 0.528 (0.278 to 0.778) | 0.653 (0.418 to 0.887) | 0.730 (0.497 to 0.962) | 0.553 (0.270 to 0.835) |

*Red highlights increases in psychological distress, relative to 2009

** Reference GHQ-12 values are adjusted for all confounders and random effects, and so do not reflect absolute values, as presented in the predicted population estimates in Figure 1A.

**Supplementary File B2 Yearly changes in psychological distress (GHQ-12 score) relative to 2009, by sex**

| **Reference value for GHQ-12 in 2009 (credible interval)**** | | |
| --- | --- | --- |
|  | **Male** | **Female** |
| **2009** | 11.64 (11.26 to 11.63) | 13.30 (13.09 to 13.50) |
| **Mean difference in GHQ-12 relative to 2009 (credible interval)** | | |
|  | **Male** | **Female** |
| **2010** | 0.089 (-0.014 to 0.191) | 0.120 (0.019 to 0.221) |
| **2011** | 0.054 (-0.050 to 0.157) | 0.176 (0.074 to 0.278) |
| **2012** | -0.017 (-0.121 to 0.088) | 0.192 (0.088 to 0.295) |
| **2013** | 0.131 (0.025 to 0.237) | 0.319 (0.215 to 0.424) |
| **2014** | 0.000 (-0.106 to 0.107) | 0.208 (0.102 to 0.313) |
| **2015** | -0.055 (-0.162 to 0.051) | 0.137 (0.032 to 0.243) |
| **2016** | 0.120 (0.013 to 0.227) | 0.295 (0.189 to 0.401) |
| **2017** | 0.308 (0.200 to 0.417) | 0.512 (0.404 to 0.620) |
| **2018** | 0.412 (0.302 to 0.522) | 0.639 (0.530 to 0.748) |
| **2019** | 0.487 (0.376 to 0.598) | 0.739 (0.629 to 0.850) |
| **2020** | 0.863 (0.750 to 0.975) | 1.430 (1.318 to 1.541) |
| **2021** | 0.887 (0.772 to 1.001) | 1.371 (1.258 to 1.485) |
| **2022** | 0.841 (0.727 to 0.956) | 1.203 (1.089 to 1.317) |
| **2023** | 1.021 (0.884 to 1.157) | 1.178 (1.042 to 1.314) |

*Red highlights increases in psychological distress, relative to 2009

** Reference GHQ-12 values are adjusted for all confounders and random effects, and so do not reflect absolute values, as presented in the predicted population estimates in Figure 1B.

**Supplementary File B3 Yearly changes in psychological distress (GHQ-12 score) relative to 2009, by ethnicity**

| **Reference value for GHQ-12 in 2009 (credible interval)** | | | | | | | | |
| --- | --- | --- | --- | --- | --- | --- | --- | --- |
|  | **South Asian** | **Asian Other** | **Black African** | **Black Caribbean** | **Mixed** | **Other** | **White Other** | **White British** |
| **2009** | 13.30 (12.76 to 13.84) | 13.45 (12.30 to 14.61) | 11.61 (10.44 to 12.79) | 11.46 (10.23 to 12.69) | 11.92 (10.80 to 13.04) | 12.96 (11.24 to 14.67) | 13.33 (12.64 to 14.03) | 11.59 (11.42 to 11.76) |
| **Mean difference in GHQ-12 relative to 2009 (credible interval)** | | | | | | | | |
|  | **South Asian** | **Asian Other** | **Black African** | **Black Caribbean** | **Mixed** | **Other** | **White Other** | **White British** |
| **2010** | 0.086 (-0.246 to 0.419) | -0.418 (-0.996 to 0.160) | 0.331 (-0.249 to 0.910) | 0.033 (-0.565 to 0.631) | -0.242 (-0.893 to 0.409) | 0.579 (-0.294 to 1.451) | 0.019 (-0.304 to 0.342) | 0.119 (0.041 to 0.198) |
| **2011** | 0.202 (-0.133 to 0.538) | -0.266 (-0.853 to 0.321) | 0.532 (-0.048 to 1.111) | 0.550 (-0.049 to 1.148) | -0.173 (-0.821 to 0.475) | 0.857 (-0.031 to 1.745) | -0.079 (-0.408 to 0.249) | 0.113 (0.034 to 0.192) |
| **2012** | 0.341 (0.000 to 0.682) | -0.145 (-0.746 to 0.456) | 0.325 (-0.270 to 0.919) | 0.148 (-0.463 to 0.760) | 0.033 (-0.621 to 0.687) | 0.418 (-0.496 to 1.332) | -0.150 (-0.487 to 0.187) | 0.087 (0.007 to 0.168) |
| **2013** | 0.289 (-0.054 to 0.632) | -0.183 (-0.804 to 0.438) | 0.099 (-0.503 to 0.701) | 0.367 (-0.254 to 0.989) | -0.483 (-1.139 to 0.172) | 0.370 (-0.559 to 1.300) | 0.043 (-0.301 to 0.388) | 0.252 (0.171 to 0.334) |
| **2014** | 0.058 (-0.285 to 0.401) | -0.307 (-0.923 to 0.310) | 0.089 (-0.520 to 0.698) | 0.159 (-0.467 to 0.784) | 0.174 (-0.481 to 0.828) | 1.116 (0.179 to 2.052) | -0.103 (-0.446 to 0.240) | 0.116 (0.034 to 0.198) |
| **2015** | -0.060 (-0.391 to 0.270) | -0.441 (-1.039 to 0.157) | -0.092 (-0.667 to 0.482) | 0.216 (-0.394 to 0.826) | 0.398 (-0.251 to 1.048) | 0.248 (-0.644 to 1.141) | -0.240 (-0.570 to 0.091) | 0.055 (-0.028 to 0.138) |
| **2016** | -0.183 (-0.511 to 0.146) | -0.251 (-0.849 to 0.347) | -0.152 (-0.736 to 0.432) | 0.297 (-0.322 to 0.916) | 0.373 (-0.276 to 1.021) | 0.892 (-0.030 to 1.814) | -0.093 (-0.424 to 0.239) | 0.257 (0.174 to 0.340) |
| **2017** | -0.097 (-0.431 to 0.237) | 0.272 (-0.348 to 0.893) | -0.205 (-0.812 to 0.402) | -0.100 (-0.733 to 0.533) | 0.399 (-0.262 to 1.061) | 0.885 (-0.059 to 1.829) | 0.037 (-0.302 to 0.376) | 0.495 (0.411 to 0.580) |
| **2018** | 0.018 (-0.321 to 0.356) | 0.425 (-0.218 to 1.068) | 0.068 (-0.553 to 0.688) | 0.413 (-0.239 to 1.065) | 0.761 (0.090 to 1.433) | 0.657 (-0.337 to 1.652) | 0.240 (-0.105 to 0.586) | 0.587 (0.502 to 0.672) |
| **2019** | 0.139 (-0.201 to 0.479) | 0.546 (-0.103 to 1.194) | 0.787 (0.152 to 1.422) | 0.442 (-0.220 to 1.104) | 1.379 (0.702 to 2.056) | 0.686 (-0.315 to 1.686) | 0.482 (0.133 to 0.831) | 0.636 (0.549 to 0.722) |
| **2020** | 0.723 (0.381 to 1.065) | 0.762 (0.109 to 1.414) | 0.944 (0.297 to 1.590) | 0.416 (-0.250 to 1.083) | 1.409 (0.731 to 2.087) | 1.741 (0.720 to 2.762) | 0.878 (0.525 to 1.231) | 1.225 (1.138 to 1.312) |
| **2021** | 0.507 (0.157 to 0.858) | 0.933 (0.264 to 1.601) | 0.890 (0.214 to 1.567) | 0.633 (-0.051 to 1.317) | 1.414 (0.726 to 2.102) | 1.820 (0.753 to 2.887) | 0.746 (0.386 to 1.106) | 1.219 (1.130 to 1.308) |
| **2022** | 0.685 (0.333 to 1.038) | 0.581 (-0.098 to 1.261) | 0.621 (-0.070 to 1.313) | 0.702 (-0.001 to 1.405) | 1.424 (0.732 to 2.116) | 2.029 (0.952 to 3.107) | 0.830 (0.472 to 1.188) | 1.062 (0.973 to 1.151) |
| **2023** | 0.543 (0.165 to 0.921) | 0.777 (0.020 to 1.534) | 0.389 (-0.383 to 1.161) | 1.234 (0.449 to 2.020) | 1.228 (0.467 to 1.989) | 1.045 (-0.121 to 2.211) | 0.787 (0.361 to 1.214) | 1.174 (1.066 to 1.282) |

*Red highlights increases in psychological distress, relative to 2009

** Reference GHQ-12 values are adjusted for all confounders and random effects, and so do not reflect absolute values, as presented in the predicted population estimates in Figure 1C.

**Supplementary File B4 Yearly changes in psychological distress (GHQ-12 score) relative to 2009, by deprivation quintile**

| **Reference value for GHQ-12 in 2009 (credible interval)**** | | | | | |
| --- | --- | --- | --- | --- | --- |
|  | **IMD quintile 1 (most deprived)** | **IMD quintile 2** | **IMD quintile 3** | **IMD quintile 4** | **IMD quintile 5 (least deprived)** |
| **2009** | 12.23 (11.88 to 12.58) | 11.98 (11.66 to 12.31) | 11.66 (11.35 to 11.98) | 11.61 (11.31 to 11.92) | 11.73 (11.43 to 12.03) |
| **Mean difference in GHQ-12 relative to 2009 (credible interval)** | | | | | |
|  | **IMD quintile 1 (most deprived)** | **IMD quintile 2** | **IMD quintile 3** | **IMD quintile 4** | **IMD quintile 5 (least deprived)** |
| **2010** | 0.019 (-0.304 to 0.342) | 0.006 (-0.161 to 0.174) | 0.319 (0.161 to 0.477) | -0.033 (-0.188 to 0.122) | 0.077 (-0.073 to 0.226) |
| **2011** | -0.079 (-0.408 to 0.249) | 0.064 (-0.106 to 0.233) | 0.157 (-0.003 to 0.317) | 0.066 (-0.090 to 0.222) | 0.032 (-0.118 to 0.183) |
| **2012** | -0.150 (-0.487 to 0.187) | -0.020 (-0.192 to 0.153) | 0.137 (-0.025 to 0.300) | -0.058 (-0.216 to 0.101) | 0.102 (-0.051 to 0.255) |
| **2013** | 0.043 (-0.301 to 0.388) | 0.163 (-0.012 to 0.338) | 0.413 (0.249 to 0.578) | 0.130 (-0.030 to 0.290) | 0.074 (-0.080 to 0.229) |
| **2014** | -0.103 (-0.446 to 0.240) | 0.045 (-0.132 to 0.221) | 0.270 (0.104 to 0.435) | 0.005 (-0.156 to 0.166) | 0.078 (-0.077 to 0.233) |
| **2015** | -0.240 (-0.570 to 0.091) | -0.092 (-0.269 to 0.084) | 0.184 (0.017 to 0.351) | -0.112 (-0.274 to 0.050) | 0.019 (-0.137 to 0.175) |
| **2016** | -0.093 (-0.424 to 0.239) | 0.072 (-0.105 to 0.250) | 0.292 (0.125 to 0.460) | 0.125 (-0.038 to 0.288) | 0.206 (0.049 to 0.362) |
| **2017** | 0.037 (-0.302 to 0.376) | 0.326 (0.146 to 0.507) | 0.472 (0.301 to 0.643) | 0.324 (0.159 to 0.489) | 0.462 (0.304 to 0.620) |
| **2018** | 0.240 (-0.105 to 0.586) | 0.332 (0.149 to 0.515) | 0.564 (0.391 to 0.737) | 0.523 (0.357 to 0.690) | 0.534 (0.374 to 0.694) |
| **2019** | 0.482 (0.133 to 0.831) | 0.554 (0.369 to 0.740) | 0.735 (0.559 to 0.910) | 0.542 (0.373 to 0.711) | 0.500 (0.339 to 0.662) |
| **2020** | 0.878 (0.525 to 1.231) | 1.002 (0.814 to 1.189) | 1.290 (1.112 to 1.467) | 1.133 (0.962 to 1.304) | 1.239 (1.077 to 1.402) |
| **2021** | 0.746 (0.386 to 1.106) | 1.031 (0.839 to 1.223) | 1.167 (0.986 to 1.348) | 1.122 (0.948 to 1.295) | 1.229 (1.064 to 1.394) |
| **2022** | 0.830 (0.472 to 1.188) | 0.939 (0.745 to 1.133) | 1.145 (0.964 to 1.326) | 0.957 (0.783 to 1.132) | 0.976 (0.811 to 1.141) |
| **2023** | 0.787 (0.361 to 1.214) | 0.801 (0.574 to 1.027) | 1.393 (1.175 to 1.611) | 1.048 (0.841 to 1.255) | 0.982 (0.781 to 1.184) |

*Red highlights increases in psychological distress, relative to 2009.

** Reference GHQ-12 values are adjusted for all confounders and random effects, and so do not reflect absolute values, as presented in the predicted population estimates in Figure 1D.

**Supplementary File B5 Yearly changes in psychological distress (GHQ-12 score) relative to 2009, by job status**

| **Reference value for GHQ-12 in 2009 (credible interval)**** | | | | | | | | |
| --- | --- | --- | --- | --- | --- | --- | --- | --- |
|  | **Employed** | **Self-employed** | **Unemployed** | **Retired** | **Parental leave** | **Family care** | **Student/training** | **Long-term sick/disabled** |
| **2009** | 12.00 (11.79 to 12.20) | 11.86 (11.33 to 11.38) | 15.17 (14.51 to 15.83) | 20.59 (15.19 to 25.99) | 12.80 (10.00 to 15.60) | 14.35 (13.54 to 15.16) | 12.17 (11.65 to 12.69) | 18.73 (18.18 to 19.28) |
| **Mean difference in GHQ-12 relative to 2009 (credible interval)** | | | | | | | | |
|  | **Employed** | **Self-employed** | **Unemployed** | **Retired** | **Parental leave** | **Family care** | **Student/training** | **Long-term sick/disabled** |
| **2010** | 0.058 (-0.045 to 0.161) | 0.155 (-0.100 to 0.411) | -0.033 (-0.445 to 0.380) | 0.209 (0.076 to 0.342) | 0.390 (-0.520 to 1.299) | -0.058 (-0.388 to 0.272) | 0.219 (-0.084 to 0.522) | 0.504 (-0.040 to 1.047) |
| **2011** | 0.057 (-0.046 to 0.161) | 0.129 (-0.129 to 0.386) | 0.415 (-0.006 to 0.836) | 0.115 (-0.020 to 0.250) | 0.250 (-0.689 to 1.188) | 0.076 (-0.259 to 0.411) | 0.575 (0.267 to 0.883) | 0.267 (-0.289 to 0.823) |
| **2012** | 0.092 (-0.013 to 0.197) | -0.106 (-0.368 to 0.155) | 0.586 (0.155 to 1.017) | -0.040 (-0.177 to 0.096) | 0.906 (-0.068 to 1.880) | 0.281 (-0.064 to 0.626) | 0.735 (0.421 to 1.049) | 0.412 (-0.154 to 0.977) |
| **2013** | 0.194 (0.088 to 0.300) | 0.114 (-0.148 to 0.377) | 0.661 (0.215 to 1.107) | 0.102 (-0.037 to 0.240) | 0.280 (-0.697 to 1.256) | 0.242 (-0.112 to 0.596) | 1.055 (0.737 to 1.374) | 0.797 (0.220 to 1.375) |
| **2014** | 0.036 (-0.071 to 0.143) | -0.111 (-0.374 to 0.152) | 0.588 (0.134 to 1.043) | 0.078 (-0.060 to 0.216) | 0.026 (-0.979 to 1.031) | 0.104 (-0.258 to 0.465) | 0.980 (0.659 to 1.302) | 0.392 (-0.195 to 0.980) |
| **2015** | 0.046 (-0.061 to 0.153) | -0.099 (-0.361 to 0.164) | 0.510 (0.055 to 0.964) | -0.054 (-0.193 to 0.085) | 0.363 (-0.622 to 1.350) | -0.012 (-0.374 to 0.351) | 0.643 (0.320 to 0.966) | 0.288 (-0.304 to 0.881) |
| **2016** | 0.139 (0.031 to 0.246) | -0.028 (-0.291 to 0.236) | 0.644 (0.180 to 1.109) | 0.211 (0.072 to 0.351) | 0.405 (-0.618 to 1.427) | 0.187 (-0.178 to 0.552) | 0.985 (0.660 to 1.310) | 0.992 (0.403 to 1.581) |
| **2017** | 0.431 (0.321 to 0.540) | 0.270 (0.003 to 0.537) | 0.807 (0.327 to 1.287) | 0.222 (0.080 to 0.363) | 0.824 (-0.199 to 1.847) | 0.393 (0.012 to 0.773) | 1.347 (1.016 to 1.679) | 0.918 (0.317 to 1.519) |
| **2018** | 0.591 (0.480 to 0.701) | 0.253 (-0.017 to 0.524) | 0.921 (0.430 to 1.412) | 0.211 (0.067 to 0.354) | 1.165 (0.162 to 2.169) | 0.750 (0.357 to 1.143) | 1.684 (1.348 to 2.021) | 1.189 (0.581 to 1.797) |
| **2019** | 0.732 (0.620 to 0.844) | 0.475 (0.201 to 0.748) | 1.435 (0.937 to 1.933) | 0.264 (0.119 to 0.408) | 1.144 (0.112 to 2.176) | 0.297 (-0.103 to 0.698) | 1.695 (1.353 to 2.037) | 0.910 (0.300 to 1.520) |
| **2020** | 1.275 (1.162 to 1.388) | 1.271 (0.992 to 1.549) | 1.595 (1.114 to 2.076) | 0.870 (0.724 to 1.016) | 1.839 (0.836 to 2.843) | 1.277 (0.858 to 1.696) | 2.293 (1.942 to 2.644) | 0.683 (0.056 to 1.311) |
| **2021** | 1.192 (1.077 to 1.307) | 1.097 (0.812 to 1.383) | 1.649 (1.165 to 2.133) | 0.934 (0.786 to 1.083) | 1.974 (0.933 to 3.015) | 0.901 (0.460 to 1.343) | 2.575 (2.212 to 2.938) | 0.741 (0.092 to 1.390) |
| **2022** | 1.097 (0.982 to 1.212) | 0.899 (0.610 to 1.188) | 1.840 (1.330 to 2.351) | 0.785 (0.636 to 0.935) | 2.016 (0.983 to 3.049) | 1.017 (0.560 to 1.475) | 2.243 (1.877 to 2.609) | 1.253 (0.615 to 1.890) |
| **2023** | 1.229 (1.092 to 1.367) | 1.014 (0.678 to 1.350) | 1.716 (1.145 to 2.288) | 0.764 (0.588 to 0.939) | 2.331 (1.116 to 3.545) | 0.811 (0.246 to 1.377) | 1.871 (1.445 to 2.298) | 1.775 (1.025 to 2.526) |

*Red highlights increases in psychological distress, relative to 2009

** Reference GHQ-12 values are adjusted for all confounders and random effects, and so do not reflect absolute values, as presented in the predicted population estimates in Figure 1E.

**Supplementary File C1 Subgroup analysis for effect of systemic shocks, by age**

|  | **Brexit Referendum** | **1st Lockdown** | **2nd Lockdown** | **Ukraine Invasion** | **Mini-Budget** |
| --- | --- | --- | --- | --- | --- |
| **Age 16 to 24** |  |  |  |  |  |
| Immediate effect | -0.142 (-0.403 to 0.119) | 0.333 (0.021 to 0.645) | 0.036 (-0.314 to 0.388) | 0.261 (-0.149 to 0.678) | -0.070 (-0.538 to 0.399) |
| Change in effect | 0.025 (0.003 to 0.048) | -0.022 (-0.046 to 0.002) | -0.033 (-0.057 to -0.008) | 0.003 (-0.033 to 0.042) | 0.005 (-0.044 to 0.058) |
| **Age 25 to 34** |  |  |  |  |  |
| Immediate effect | 0.216 (-0.012 to 0.444) | 0.442 (0.156 to 0.727) | -0.167 (-0.517 to 0.181) | -0.244 (-0.594 to 0.107) | 0.274 (-0.130 to 0.677) |
| Change in effect | 0.011 (-0.007 to 0.029) | -0.034 (-0.056 to -0.012) | -0.030 (-0.055 to -0.006) | 0.018 (-0.012 to 0.049) | -0.002 (-0.043 to 0.040) |
| **Age 35 to 44** |  |  |  |  |  |
| Immediate effect | -0.058 (-0.271 to 0.153) | 0.620 (0.374 to 0.872) | -0.328 (-0.664 to 0.004) | -0.019 (-0.368 to 0.345) | 0.230 (-0.133 to 0.593) |
| Change in effect | 0.005 (-0.014 to 0.024) | -0.025 (-0.044 to -0.007) | -0.034 (-0.057 to -0.011) | 0.020 (-0.011 to 0.051) | 0.004 (-0.039 to 0.046) |
| **Age 45 to 54** |  |  |  |  |  |
| Immediate effect | 0.281 (0.085 to 0.477) | 0.694 (0.474 to 0.917) | -0.237 (-0.574 to 0.089) | -0.162 (-0.488 to 0.172) | -0.105 (-0.434 to 0.225) |
| Change in effect | 0.007 (-0.011 to 0.024) | -0.034 (-0.052 to -0.017) | -0.049 (-0.071 to -0.027) | 0.003 (-0.028 to 0.033) | -0.000 (-0.042 to 0.041) |
| **Age 55 to 64** |  |  |  |  |  |
| Immediate effect | 0.184 (-0.013 to 0.380) | 0.747 (0.519 to 0.975) | -0.250 (-0.544 to 0.044) | 0.002 (-0.289 to 0.309) | -0.214 (-0.512 to 0.085) |
| Change in effect | 0.023 (0.005 to 0.041) | -0.020 (-0.039 to -0.001) | -0.029 (-0.049 to -0.009) | 0.010 (-0.018 to 0.037) | 0.022 (-0.015 to 0.059) |
| **Age 65 to 74** |  |  |  |  |  |
| Immediate effect | 0.089 (-0.095 to 0.271) | 0.821 (0.611 to 1.031) | -0.170 (-0.479 to 0.128) | -0.046 (-0.319 to 0.243) | -0.109 (-0.384 to 0.166) |
| Change in effect | -0.015 (-0.032 to 0.002) | -0.049 (-0.067 to -0.032) | -0.059 (-0.079 to -0.040) | 0.003 (-0.023 to 0.028) | 0.016 (-0.021 to 0.052) |
| **Age 75+** |  |  |  |  |  |
| Immediate effect | 0.197 (-0.045 to 0.438) | 0.736 (0.524 to 0.947) | 0.142 (-0.233 to 0.526) | -0.061 (-0.346 to 0.223) | -0.374 (-0.708 to -0.040) |
| Change in effect | -0.017 (-0.039 to 0.005) | -0.016 (-0.031 to -0.002) | -0.054 (-0.078 to -0.030) | -0.025 (-0.050 to 0.001) | 0.016 (-0.027 to 0.062) |

*Red highlights increases in psychological distress and blue highlights decreases in psychological distress, after each systemic shock. The immediate effect describes a direct effect of the shock, while the change in effect shows any gradual change over time in the period following the shock.

**Supplementary File C2 Subgroup analysis for effect of systemic shocks, by sex**

|  | **Brexit Referendum** | **1st Lockdown** | **2nd Lockdown** | **Ukraine Invasion** | **Mini-Budget** |
| --- | --- | --- | --- | --- | --- |
| **Male** |  |  |  |  |  |
| Immediate effect | 0.136 (0.016 to 0.255) | 0.385 (0.231 to 0.540) | -0.102 (-0.327 to 0.125) | -0.055 (-0.245 to 0.138) | 0.031 (-0.154 to 0.216) |
| Change in effect | 0.002 (-0.008 to 0.012) | -0.015 (-0.027 to -0.003) | -0.021 (-0.034 to -0.008) | 0.016 (-0.001 to 0.034) | 0.009 (-0.014 to 0.032) |
| **Female** |  |  |  |  |  |
| Immediate effect | 0.121 (0.002 to 0.243) | 0.862 (0.712 to 1.011) | -0.227 (-0.450 to -0.010) | 0.010 (-0.176 to 0.198) | -0.141 (-0.325 to 0.043) |
| Change in effect | 0.009 (-0.001 to 0.019) | -0.038 (-0.050 to -0.027) | -0.046 (-0.058 to -0.033) | 0.006 (-0.011 to 0.023) | 0.019 (-0.005 to 0.042) |

*Red highlights increases in psychological distress and blue highlights decreases in psychological distress, after each systemic shock. The immediate effect describes a direct effect of the shock, while the change in effect shows any gradual change over time in the period following the shock.

**Supplementary File C3 Subgroup analysis for effect of systemic shocks, by ethnicity**

|  | **Brexit Referendum** | **1st Lockdown** | **2nd Lockdown** | **Ukraine Invasion** | **Mini-Budget** |
| --- | --- | --- | --- | --- | --- |
| **South Asian** |  |  |  |  |  |
| Immediate effect | 0.208 (-0.076 to 0.509) | 0.453 (0.154 to 0.749) | -0.254 (-0.632 to 0.124) | 0.121 (-0.339 to 0.580) | -0.140 (-0.611 to 0.331) |
| Change in effect | 0.018 (-0.006 to 0.040) | -0.023 (-0.044 to -0.004) | -0.023 (-0.050 to 0.003) | 0.008 (-0.030 to 0.048) | 0.010 (-0.039 to 0.059) |
| **Asian Other** |  |  |  |  |  |
| Immediate effect | 0.230 (-0.327 to 0.728) | 0.523 (-0.101 to 1.147) | 0.026 (-0.658 to 0.712) | -0.215 (-1.086 to 0.656) | -0.130 (-1.201 to 0.942) |
| Change in effect | 0.019 (-0.012 to 0.056) | -0.017 (-0.052 to 0.020) | -0.021 (-0.062 to 0.023) | -0.004 (-0.065 to 0.057) | -0.000 (-0.097 to 0.096) |
| **Black African** |  |  |  |  |  |
| Immediate effect | -0.013 (-0.492 to 0.472) | 0.313 (-0.369 to 0.953) | -0.118 (-0.842 to 0.599) | 0.253 (-0.661 to 1.186) | -0.251 (-1.317 to 0.817) |
| Change in effect | 0.020 (-0.015 to 0.054) | -0.048 (-0.088 to -0.011) | -0.049 (-0.094 to -0.004) | -0.026 (-0.095 to 0.048) | -0.038 (-0.129 to 0.055) |
| **Black Caribbean** |  |  |  |  |  |
| Immediate effect | 0.083 (-0.426 to 0.697) | -0.105 (-0.735 to 0.520) | 0.073 (-0.709 to 0.766) | 0.109 (-0.770 to 0.978) | 0.005 (-1.088 to 1.094) |
| Change in effect | -0.004 (-0.054 to 0.023) | -0.010 (-0.057 to 0.025) | -0.021 (-0.100 to 0.022) | -0.015 (-0.078 to 0.045) | -0.019 (-0.118 to 0.078) |
| **Mixed** |  |  |  |  |  |
| Immediate effect | 0.072 (-0.474 to 0.616) | 0.151 (-0.488 to 0.757) | -0.138 (-0.825 to 0.545) | 0.160 (-0.688 to 1.032) | -0.389 (-1.355 to 0.582) |
| Change in effect | 0.007 (-0.033 to 0.044) | -0.024 (-0.062 to 0.014) | -0.010 (-0.049 to 0.044) | 0.034 (-0.037 to 0.110) | 0.024 (-0.063 to 0.113) |
| **Other** |  |  |  |  |  |
| Immediate effect | 0.815 (0.054 to 1.786) | 1.533 (0.511 to 2.539) | 0.925 (-0.256 to 1.977) | 0.773 (-0.667 to 2.187) | 2.020 (0.405 to 3.627) |
| Change in effect | -0.013 (-0.087 to 0.043) | -0.031 (-0.084 to 0.023) | -0.057 (-0.115 to -0.004) | -0.099 (-0.191 to -0.006) | -0.252 (-0.388 to -0.117) |
| **White British** |  |  |  |  |  |
| Immediate effect | -0.069 (-0.378 to 0.241) | 0.701 (0.573 to 0.828) | -0.177 (-0.457 to 0.103) | -0.204 (-0.631 to 0.200) | -0.052 (-0.200 to 0.095) |
| Change in effect | 0.006 (-0.017 to 0.029) | -0.028 (-0.038 to -0.018) | -0.036 (-0.046 to -0.025) | -0.028 (-0.057 to -0.002) | 0.018 (-0.002 to 0.039) |
| **White Other** |  |  |  |  |  |
| Immediate effect | 0.082 (-0.015 to 0.179) | 0.571 (0.212 to 0.930) | -0.204 (-0.631 to 0.200) | 0.136 (-0.329 to 0.612) | -0.568 (-1.136 to -0.001) |
| Change in effect | 0.008 (-0.000 to 0.016) | -0.024 (-0.048 to -0.002) | -0.028 (-0.057 to -0.002) | -0.016 (-0.052 to 0.022) | 0.025 (-0.031 to 0.081) |

*Red highlights increases in psychological distress and blue highlights decreases in psychological distress, after each systemic shock. The immediate effect describes a direct effect of the shock, while the change in effect shows any gradual change over time in the period following the shock.

**Supplementary File C4 Subgroup analysis for effect of systemic shocks, by deprivation quintile**

|  | **Brexit Referendum** | **1st Lockdown** | **2nd Lockdown** | **Ukraine Invasion** | **Mini-Budget** |
| --- | --- | --- | --- | --- | --- |
| **IMD 1: Most deprived** |  |  |  |  |  |
| Immediate effect | 0.200 (0.002 to 0.404) | 0.254 (0.003 to 0.498) | -0.130 (-0.531 to 0.266) | 0.308 (-0.037 to 0.664) | 0.005 (-0.338 to 0.347) |
| Change in effect | 0.006 (-0.012 to 0.024) | -0.012 (-0.031 to 0.006) | -0.009 (-0.033 to 0.015) | 0.017 (-0.013 to 0.049) | 0.025 (-0.015 to 0.066) |
| **IMD 2** |  |  |  |  |  |
| Immediate effect | 0.212 (0.017 to 0.404) | 0.578 (0.362 to 0.795) | -0.030 (-0.347 to 0.261) | -0.069 (-0.358 to 0.220) | -0.035 (-0.347 to 0.278) |
| Change in effect | 0.009 (-0.007 to 0.025) | -0.033 (-0.050 to -0.017) | -0.043 (-0.063 to -0.024) | 0.003 (-0.023 to 0.029) | -0.009 (-0.045 to 0.027) |
| **IMD 3** |  |  |  |  |  |
| Immediate effect | 0.185 (0.004 to 0.374) | 0.693 (0.444 to 0.949) | -0.381 (-0.749 to -0.022) | -0.120 (-0.353 to 0.114) | -0.211 (-0.507 to 0.084) |
| Change in effect | 0.025 (0.008 to 0.041) | -0.019 (-0.039 to 0.002) | -0.028 (-0.048 to -0.007) | 0.011 (-0.009 to 0.030) | 0.026 (-0.014 to 0.066) |
| **IMD 4** |  |  |  |  |  |
| Immediate effect | 0.080 (-0.100 to 0.256) | 0.735 (0.524 to 0.950) | -0.101 (-0.374 to 0.172) | -0.193 (-0.468 to 0.086) | 0.007 (-0.269 to 0.283) |
| Change in effect | 0.004 (-0.011 to 0.019) | -0.029 (-0.046 to -0.013) | -0.044 (-0.062 to -0.026) | 0.004 (-0.022 to 0.029) | 0.003 (-0.032 to 0.038) |
| **IMD 5: Least deprived** |  |  |  |  |  |
| Immediate effect | 0.016 (-0.160 to 0.193) | 0.932 (0.754 to 1.112) | -0.163 (-0.435 to 0.098) | -0.126 (-0.394 to 0.146) | 0.028 (-0.231 to 0.288) |
| Change in effect | 0.000 (-0.015 to 0.015) | -0.027 (-0.041 to -0.014) | -0.044 (-0.061 to -0.027) | 0.018 (-0.007 to 0.043) | 0.021 (-0.014 to 0.056) |

*Red highlights increases in psychological distress and blue highlights decreases in psychological distress, after each systemic shock. The immediate effect describes a direct effect of the shock, while the change in effect shows any gradual change over time in the period following the shock.

**Supplementary File C5 Subgroup analysis for effect of systemic shocks, by employment status**

|  | **Brexit Referendum** | **1st Lockdown** | **2nd Lockdown** | **Ukraine Invasion** | **Mini-Budget** |
| --- | --- | --- | --- | --- | --- |
| **Self-employed** |  |  |  |  |  |
| Immediate effect | 0.056 (-0.067 to 0.179) | 0.547 (0.387 to 0.705) | -0.309 (-0.662 to -0.058) | -0.081 (-0.263 to 0.102) | 0.036 (-0.149 to 0.221) |
| Change in effect | 0.013 (0.003 to 0.024) | -0.028 (-0.040 to -0.015) | -0.033 (-0.046 to -0.020) | 0.009 (-0.007 to 0.026) | 0.001 (-0.022 to 0.025) |
| **Employed** |  |  |  |  |  |
| Immediate effect | 0.123 (-0.151 to 0.397) | 0.860 (0.563 to 1.155) | -0.374 (-0.815 to 0.040) | 0.184 (-0.285 to 0.665) | -0.074 (-0.536 to 0.388) |
| Change in effect | 0.013 (-0.010 to 0.037) | -0.032 (-0.055 to -0.011) | -0.029 (-0.057 to 0.001) | 0.034 (-0.008 to 0.077) | 0.035 (-0.021 to 0.091) |
| **Unemployed** |  |  |  |  |  |
| Immediate effect | -0.042 (-0.517 to 0.435) | -0.060 (-0.595 to 0.473) | 0.036 (-0.505 to 0.575) | 0.057 (-0.621 to 0.736) | 0.516 (-0.377 to 1.411) |
| Change in effect | 0.014 (-0.018 to 0.046) | -0.018 (-0.053 to 0.016) | -0.018 (-0.051 to 0.015) | -0.002 (-0.049 to 0.047) | -0.014 (-0.097 to 0.070) |
| **Retired** |  |  |  |  |  |
| Immediate effect | 0.177 (0.031 to 0.322) | 0.992 (0.808 to 1.179) | -0.078 (-0.445 to 0.298) | -0.040 (-0.274 to 0.196) | -0.255 (-0.470 to -0.039) |
| Change in effect | -0.010 (-0.023 to 0.003) | -0.043 (-0.058 to -0.028) | -0.057 (-0.073 to -0.041) | -0.000 (-0.022 to 0.021) | 0.027 (-0.005 to 0.058) |
| **Parental leave** |  |  |  |  |  |
| Immediate effect | -0.260 (-1.276 to 0.602) | 0.688 (-0.334 to 1.683) | 0.248 (-0.888 to 1.356) | 0.403 (-1.028 to 1.826) | 0.004 (-1.845 to 1.850) |
| Change in effect | 0.023 (-0.025 to 0.068) | -0.010 (-0.061 to 0.039) | -0.009 (-0.068 to 0.052) | -0.028 (-0.128 to 0.072) | -0.040 (-0.210 to 0.130) |
| **Family care** |  |  |  |  |  |
| Immediate effect | 0.391 (0.021 to 0.771) | 1.029 (0.542 to 1.545) | -0.237 (-1.015 to 0.316) | 0.197 (-0.489 to 0.903) | -0.527 (-1.385 to 0.330) |
| Change in effect | -0.004 (-0.035 to 0.024) | -0.032 (-0.070 to -0.000) | -0.025 (-0.062 to 0.013) | -0.030 (-0.082 to 0.026) | -0.002 (-0.084 to 0.081) |
| **Student/training** |  |  |  |  |  |
| Immediate effect | 0.125 (-0.228 to 0.468) | 0.742 (0.348 to 1.134) | 0.375 (-0.045 to 0.791) | -0.218 (-0.731 to 0.307) | -0.280 (-0.893 to 0.335) |
| Change in effect | 0.005 (-0.026 to 0.034) | -0.036 (-0.065 to -0.008) | -0.061 (-0.089 to -0.034) | -0.026 (-0.067 to 0.023) | 0.001 (-0.065 to 0.071) |
| **Long-term sick/disabled** |  |  |  |  |  |
| Immediate effect | 0.793 (0.245 to 1.358) | -0.324 (-0.987 to 0.460) | -0.200 (-0.872 to 0.540) | 0.758 (-0.085 to 1.607) | 0.159 (-0.858 to 1.171) |
| Change in effect | -0.025 (-0.069 to 0.011) | 0.017 (-0.033 to 0.060) | 0.025 (-0.024 to 0.066) | -0.006 (-0.075 to 0.061) | -0.004 (-0.106 to 0.096) |

*Red highlights increases in psychological distress and blue highlights decreases in psychological distress, after each systemic shock. The immediate effect describes a direct effect of the shock, while the change in effect shows any gradual change over time in the period following the shock.

**Supplementary File D1 Results of difference in pre/post shock analysis showing effect of systemic shocks, by age**

| **Age group (years)** | **Pre-Brexit** | **After Brexit referendum** | **After 1^st^ Covid lockdown** | **After 2nd Covid lockdown** | **After Ukraine Invasion** | **After mini-budget** |
| --- | --- | --- | --- | --- | --- | --- |
| **16-24** | Reference | -0.080 (-0.323 to 0.160) | 0.220 (-0.070 to 0.509) | -0.020 (-0.373 to 0.347) | 0.222 (-0.134 to 0.589) | -0.161 (-0.531 to 0.212) |
| **25-34** | Reference | 0.255 (0.018 to 0.492) | 0.318 (0.030 to 0.605) | -0.166 (-0.534 to 0.204) | -0.151 (-0.483 to 0.182) | 0.374 (0.034 to 0.714) |
| **35-44** | Reference | 0.020 (-0.179 to 0.213) | 0.577 (0.333 to 0.814) | -0.346 (-0.640 to -0.035) | 0.019 (-0.270 to 0.321) | 0.221 (-0.072 to 0.520) |
| **45-54** | Reference | 0.313 (0.130 to 0.494) | 0.546 (0.326 to 0.760) | -0.341 (-0.616 to -0.060) | -0.143 (-0.400 to 0.120) | -0.059 (-0.325 to 0.206) |
| **55-64** | Reference | 0.237 (0.051 to 0.425) | 0.672 (0.461 to 0.881) | -0.280 (-0.571 to 0.054) | 0.104 (-0.147 to 0.375) | -0.106 (-0.356 to 0.140) |
| **65-75** | Reference | 0.082 (-0.100 to 0.257) | 0.559 (0.340 to 0.765) | -0.229 (-0.535 to 0.074) | -0.014 (-0.249 to 0.242) | -0.057 (-0.287 to 0.172) |
| **75+** | Reference | 0.229 (0.014 to 0.431) | 0.604 (0.371 to 0.826) | 0.035 (-0.243 to 0.305) | 0.009 (-0.251 to 0.264) | -0.286 (-0.548 to -0.025) |

**Supplementary File D2 Results of pre/post shock analysis showing effect of systemic shocks, by sex**

| **Sex** | **Pre-Brexit** | **After Brexit referendum** | **After 1^st^ Covid lockdown** | **After 2nd Covid lockdown** | **After Ukraine Invasion** | **After Truss budget** |
| --- | --- | --- | --- | --- | --- | --- |
| **Male** | Reference | 0.139 (0.022 to 0.256) | 0.304 (0.166 to 0.441) | -0.104 (-0.323 to 0.128) | 0.022 (-0.141 to 0.191) | 0.062 (-0.096 to 0.219) |
| **Female** | Reference | 0.161 (0.040 to 0.282) | 0.624 (0.478 to 0.769) | -0.177 (-0.558 to 0.208) | 0.171 (-0.007 to 0.350) | -0.088 (-0.252 to 0.076) |

**Supplementary File D3 Results of pre/post shock analysis showing effect of systemic shocks, by ethnicity**

| **Ethnicity** | **Pre-Brexit** | **After Brexit referendum** | **After 1^st^ Covid lockdown** | **After 2nd Covid lockdown** | **After Ukraine Invasion** | **After mini-budget** |
| --- | --- | --- | --- | --- | --- | --- |
| South Asian | Reference | 0.250 (-0.027 to 0.529) | 0.339 (0.019 to 0.654) | -0.322 (-0.695 to 0.052) | 0.139 (-0.239 to 0.518) | -0.204 (-0.584 to 0.178) |
| Asian (Other) | Reference | 0.280 (-0.299 to 0.840) | 0.660 (-0.030 to 1.360) | -0.075 (-0.878 to 0.768) | 0.022 (-0.806 to 0.868) | 0.172 (-0.705 to 1.060) |
| Black African | Reference | -0.007 (-0.509 to 0.513) | -0.063 (-0.751 to 0.603) | -0.345 (-1.103 to 0.451) | 0.083 (-0.738 to 0.945) | -0.651 (-1.513 to 0.234) |
| Black Caribbean | Reference | 0.112 (-0.447 to 0.707) | -0.305 (-0.992 to 0.374) | -0.077 (-0.908 to 0.691) | -0.141 (-0.986 to 0.677) | -0.327 (-1.216 to 0.539) |
| Mixed | Reference | 0.098 (-0.508 to 0.738) | 0.034 (-0.657 to 0.726) | -0.072 (-0.916 to 0.887) | 0.502 (-0.319 to 1.382) | -0.190 (-1.007 to 0.646) |
| Other | Reference | 0.977 (0.145 to 1.860) | 1.525 (0.414 to 2.616) | 0.214 (-1.020 to 1.488) | 0.077 (-1.284 to 1.477) | 0.533 (-0.909 to 2.064) |
| White Other | Reference | -0.089 (-0.438 to 0.253) | 0.514 (0.107 to 0.929) | -0.378 (-0.904 to 0.182) | 0.289 (-0.179 to 0.767) | -0.496 (-0.972 to -0.024) |
| White British | Reference | 0.113 (0.016 to 0.209) | 0.525 (0.408 to 0.641) | -0.160 (-0.513 to 0.196) | 0.094 (-0.049 to 0.237) | -0.003 (-0.135 to 0.130) |

**Supplementary File D4 Results of pre/post shock analysis showing effect of systemic shocks, by deprivation quintile**

| **IMD quintile** | **Pre-Brexit** | **After Brexit referendum** | **After 1^st^ Covid lockdown** | **After 2nd Covid lockdown** | **After Ukraine Invasion** | **After mini-budget** |
| --- | --- | --- | --- | --- | --- | --- |
| 1 (most deprived) | Reference | 0.205 (0.012 to 0.399) | 0.232 (-0.022 to 0.474) | -0.035 (-0.361 to 0.313) | 0.355 (0.062 to 0.666) | 0.033 (-0.253 to 0.322) |
| 2 | Reference | 0.265 (0.086 to 0.440) | 0.445 (0.230 to 0.648) | -0.129 (-0.381 to 0.162) | -0.034 (-0.274 to 0.225) | -0.099 (-0.349 to 0.161) |
| 3 | Reference | 0.245 (0.063 to 0.427) | 0.608 (0.393 to 0.823) | -0.372 (-0.713 to -0.030) | 0.137 (-0.118 to 0.392) | -0.105 (-0.351 to 0.141) |
| 4 | Reference | 0.155 (-0.001 to 0.311) | 0.589 (0.410 to 0.767) | -0.209 (-0.418 to 0.001) | -0.188 (-0.393 to 0.018) | 0.046 (-0.168 to 0.260) |
| 5 (least deprived) | Reference | 0.077 (-0.093 to 0.247) | 0.783 (0.587 to 0.975) | -0.199 (-0.511 to 0.112) | -0.006 (-0.230 to 0.225) | 0.153 (-0.066 to 0.371) |

**Supplementary File D5 Results of pre/post shock analysis model showing effect of systemic shocks, by job status**

| **Job Status** | **Pre-Brexit** | **After Brexit referendum** | **After 1^st^ Covid lockdown** | **After 2nd Covid lockdown** | **After Ukraine Invasion** | **After mini-budget** |
| --- | --- | --- | --- | --- | --- | --- |
| **Employed** | Reference | 0.143 (0.027 to 0.258) | 0.443 (0.307 to 0.578) | -0.311 (-0.510 to -0.112) | -0.025 (-0.183 to 0.135) | 0.088 (-0.066 to 0.244) |
| **Self-employed** | Reference | 0.154 (-0.108 to 0.411) | 0.729 (0.405 to 1.041) | -0.395 (-0.814 to 0.076) | 0.236 (-0.173 to 0.658) | -0.003 (-0.381 to 0.380) |
| **Unemployed** | Reference | -0.077 (-0.551 to 0.396) | -0.179 (-0.735 to 0.369) | 0.107 (-0.480 to 0.707) | -0.020 (-0.656 to 0.627) | 0.243 (-0.438 to 0.941) |
| **Retired** | Reference | 0.164 (0.019 to 0.309) | 0.730 (0.563 to 0.896) | -0.124 (-0.459 to 0.214) | 0.045 (-0.155 to 0.246) | -0.153 (-0.338 to 0.033) |
| **Parental leave** | Reference | -0.067 (-1.179 to 0.847) | 0.651 (-0.433 to 1.682) | -0.121 (-1.270 to 1.066) | 0.433 (-0.808 to 1.697) | -0.405 (-1.725 to 0.923) |
| **Family care** | Reference | 0.402 (0.062 to 0.769) | 0.988 (0.521 to 1.460) | -0.577 (-1.141 to -0.008) | 0.376 (-0.232 to 0.988) | -0.574 (-1.225 to 0.076) |
| **Student/training** | Reference | 0.122 (-0.226 to 0.442) | 0.427 (0.035 to 0.814) | 0.105 (-0.363 to 0.608) | -0.232 (-0.704 to 0.262) | -0.345 (-0.833 to 0.152) |
| **Long-term sick or disabled** | Reference | 0.743 (0.225 to 1.263) | -0.051 (-0.675 to 0.574) | 0.052 (-0.632 to 0.736) | 0.808 (0.101 to 1.515) | -0.071 (-0.816 to 0.674) |
